# Developmental and Psychometric Properties of a Belief-based Reproductive Health Behavior Questionnaire for Female Adolescents

**Published:** 2018-08

**Authors:** Fatemeh DARABI, Mehdi YASERI, Hossein SAFARI, Mohammad Hossein KAVEH, Farideh KHALAJ ABADI FARAHANI, Davoud SHOJAEIZADEH

**Affiliations:** 1. Dept. of Public Health, Asadabad School of Medical Sciences, Asadabad, Iran; 2. Dept. of Epidemiology and Biostatistics, School of Public Health, Tehran University of Medical Sciences, Tehran, Iran; 3. Dept. of Health Management and Economics Research Center, Iran University of Medical Sciences, Tehran, Iran; 4. Dept. of Health Education and Health Promotion, School of Health, Shiraz University of Medical Sciences, Shiraz, Iran; 5. Dept. of Population, Health and Family Planning, National Institute for Population Research, Tehran, Iran; 6. Dept. of Education Health and Promotion Health, School of Public Health, Tehran University of Medical Sciences, Tehran, Iran

**Keywords:** Psychometric, Questionnaire, Reliability, Validity, Reproductive health, Adolescent

## Abstract

**Background:**

The aim of this study was to develop and evaluate the psychometric properties of a questionnaire for the measurement of reproductive health among female adolescents in Iran.

**Methods:**

This cross-sectional study was conducted among 289 female students aged 12–15 yr in Tehran, Iran from 2015–2016. The participants were selected using multi-stage random cluster sampling. In addition, the Belief-Based Reproductive Health Questionnaire (BBRHQ) was designed using the properties of the Theory of Planned Behavior (TPB).

**Results:**

Exploratory factor analysis of 104 items identified a six-factor solution. These factors jointly accounted for 67% of the observed variance of outcome variable. The confirmatory factor analysis indicated a good fit to the data. In addition, the Cronbach’s alpha coefficient showed an excellent internal consistency (alpha=0.92).

**Conclusion:**

Belief-Based Reproductive Health Questionnaire (BBRHQ) is a valid and reliable instrument for measurement of reproductive health behaviors of adolescents.

## Introduction

Reproductive health is “a state of complete physical, mental, and social well-being and not merely the absence of disease or infirmity in all matters relating to the reproductive system and to its functions and processes” (1). Since more than half of the young people in the world start up their sexual activity during their adolescence years, therefore, reproductive health of youth is an important issue which requires a lot of attention (2–4).

In addition, girls compared to boys suffer from poor knowledge and information on sexual and reproductive health (5). Hence, it is high time to invest in adolescent’s reproductive health. Furthermore, a suitable place to reach this goal can be the school setting as it provides easier access to many young people as well as their parents (6). The theoretical underpinning of this research is the theory of planned behavior (TPB) in order to predict reproductive health among female students (7). In line with this, several potential distal variables such as socio-demographic variables (8) including age, and locus of control (9), and parental supervision (10) have been proposed which may influence the behavior (11). Perceived parental control may improve children’s behaviors (12).

There are a few studies (13) assessing the direct measures of TPB so as to predict reproductive health and behaviors of adolescents (14). Therefore, valid and reliable instruments are needed to assess sexual and reproductive health of adolescents based on behavior change theories of social and behavioral sciences.

In the current study, we investigated the influence of perceived parental control within the TPB.

## Materials and Methods

### Design and sampling

This cross-sectional study was conducted among 289 female students aged 12–15 yr in Tehran, Iran from 2015–2016. The adolescents were recruited from 6 female schools in three districts in Tehran, Iran. The schools were selected from among 26 female middle schools in three districts of Tehran (districts 2, 4 and 10), which comprise a mixture of working-class and lower-middle-class families in one of Tehran’s most populated inner-city areas. From each school, three classes were selected randomly; one class from each grade. Therefore, 18 classes were enrolled in this study.

### Survey Instruments

A self-administered questionnaire was developed using direct measures of TPB theory (15). Some parts of this instrument were based on the questionnaire developed by the WHO (16). Other sections of our instrument were developed based on the literature review and a qualitative study (eight focus-group discussions with 40 participants). The final questionnaire consisted of 145 questions and items.

### Measures

#### 1. Socio-economic and demographic characteristics

Thirteen items were included in the questionnaire to elicit personal information on age, place of residence, etc.

#### 2. Reproductive Health Knowledge

Here, 28 items were used and each was scored using 3 categories (true, false, and do not know). Each correct answer was given score one, and wrong and “do not know” responses were scored zero.

#### 3. TPB constructs

##### 1.3 Attitude towards reproductive health behavior

Twenty-one items regarding attitude towards reproductive health, derived from relevant literature, were employed.

##### 2.3 Subjective norms of reproductive health behavior

Nineteen items were used to assess the influence of important people (parents …) on their opinion about reproductive health.

##### 3.3 Perceived Behavioral Control (PBC) of reproductive health behavior

Twenty-five items were used to measure the students’ perceptions of behavioral control about behaving in a way that preserves their reproductive health. Answers were rated on a 5-point Likert differential scale ranging from 1 (very difficult) to 5 (very easy). PBC questions were designed (7, 17).

##### 4.3 Perceived parental control over reproductive health behavior

Eight items were used to assess this aspect of the study (18).

##### 5.3 Behavioral intention to reproductive health behavior

Eighteen items about attitude towards reproductive health, derived from relevant literature, were employed to measure this item.

##### 6.3 Reproductive health skills and behaviors

Thirteen items were used to evaluate reproductive health skills. Items of the questionnaire reversed included items 2,9,10,11,14,15,16,19,20,21,34 and 36. The maximum total point was 100.

### Face Validity

Ten adolescent girls aged 12–15 were recruited using convenience sampling to determine the ambiguity, relevance, and difficulty of each item. At this stage, none of the items was removed, but 3 items were changed as a result of the adolescents’ suggestions.

### Content Validity

First, 10 specialists in health education and health promotion, public health, and reproductive health areas reviewed the questionnaire to check its grammar, wording, item allocation, and scaling. In order to calculate the CVR, 13 other specialists were asked to assess each item on a 3-point Likert scale (1= were essential, 2= were useful but not essential, 3= were not essential) during the quantitative stage. Then, based on Lawshe’s table (19), items that scored greater than or equal to 0.54 were kept on the scale. Throughout this phase, 27 items were removed. In order to calculate the CVI, 10 additional expert panelists were asked to determine the relevance, clarity, and simplicity of each item using a 4-point Likert scale. In addition, two separate groups of experts were used for more precision. However, in accordance with Waltz and Baussel (20), items with CVI value greater than or equal to 0.79 were accepted, and 17 items that did not meet this criterion were deleted. 104 items had a CVI value of greater than or equal to 0.79.

### Construct Validity

This validity was investigated using exploratory factor analysis with varimax rotation. The Kaiser-Meyer-Olkin (KMO) and Bartlett’s Test of Sphericity were used to assess the appropriateness of the sample for the factor analysis. Eigenvalues above 1 and scree plot were used to determine the number of factors. Factor loadings equal to or greater than 0.3 were considered appropriate (21). Data were analyzed using SPSS version 23.0 (Chicago, IL, USA) as well as AMOS 23.

### Reliability

Internal consistency was evaluated by Cronbach’s α coefficient (22). A sub-sample of students (n=45) completed the questionnaire twice with a 2-wk interval in order to examine the stability of the scale by calculating Intra-class Correlation Coefficient (ICC) where the ICC of 0.4 or above was considered acceptable(23) (Table 1).

**Table 1:** Mean, number of items, and intra-class coefficient of Iranian students' reproductive health questionnaire (BBRHQ) constructs (n = 289)

***Subscale***	***Number of items***	***Mean ± SD***	***ICC (n = 289)***
Reproductive health Knowledge	28	53.05±18.97	0.91
Attitudes towards reproductive health	21	24.88±9.91	0.96
Subjective Norm	19	23.80±11.26	0.97
Behavioral Intention	18	25.66±9.40	0.96
Perceived parental control	8	25.45±13.02	0.96
Perceived behavioral control	25	24.57±9.98	0.96
Reproductive health Behavior	13	26.96±11.34	0.95
Total	132	25.04±4.40	0.86

### Ethical approval

The Ethics Committee of Tehran University of Medical Sciences approved the study (Ethics code No. 651, dated Monday, April 25, 2016).

## Results

The socioeconomic and demographic characteristics of the studied participants are shown in Table 2.

**Table 2:** Socio-economic and demographic characteristics of female adolescents (n = 289)

***Variable***	***No. (%)***
**Age (mean, SD)**	14.26(0.955)
**High school Grade**	
First	96(33.2%)
Second	97(33.6%)
Third	96(33.2%)
**Economic situation**	
Very good	23(8%)
Good	128(44.3%)
Average	123(42.6%)
Weak	15(5.2%)
**Mother’ education**	
Illiterate	2 (0.7%)
Under diploma	72(25.1%)
Diploma	138(48.1)
University degree	75 (26.1%)
**Father’ education**	
Illiterate	2 (0.7%)
Under diploma	77(27%)
Diploma	119(41.8%)
University degree	87(30.5 %)
**Mother’s employment**	
Housewife	208(72%)
Employed	79(27.3%)
**Father’s employment**	
Employed	269(93.1%)
Unemployed	20(6.9%)

### Validity

In the qualitative face validity, participants stated that they have had no problems in reading and understanding the items. In the quantitative content validity phase, items with CVR and CVI less than 0.62 and 0.80 were respectively omitted (24 items). The mean of content validity ratio was 0.64.

Moreover, the mean of the content validity index (CVI) was 0.74. Exploratory factor analysis (EFA) was used to evaluate construct validity (Table 3).

**Table 3:** The results obtained from exploratory factor analysis with varimax rotation among adolescents aged 12–15 (n = 289)

***Items***	***Factors***						
	**1**	**2**	**3**	**4**	**5**	**6**
Despite a lot of homework, I’m sure that I can have a proper diet.	0.909					
I am confident that I can stay away from contaminated objects such as syringes and needles infected with HIV/AIDS virus.	0.895					
Although I am lazy to do some exercise, I can take a half an hour walk every day.	0.894					
While eating fast food with my friends is a pleasure for me, but I can use homemade and fresh food.	0.876					
Although I like to spend much time underwater in bathroom or swim in a pool, I can have a standing bath during my menstruation.	0.870					
Daily consumption of 2 to 3 servings of dairy products is easy for me.	0.868					
I am confident that I can consume enough vegetables (such as lettuce, cabbage, cucumber, carrot, broccoli …) for 3 to 5 servings per day.	0.863					
It is easy for me to control changes in my mood such as depression and anxiety during my menstruation.	0.830					
I am sure that I can I exercise at least 30 to 60 minutes a day despite having little time.	0.830					
Flexibility and ability to communicate effectively with others is easy for me during adolescence.	0.829					
I’m sure that I can do more social activities during my adolescence.	0.827					
I am confident that I can consume 2 to 3 servings of dairy products per day.	0.825					
I’m sure that I can avoid risky behaviors such as unprotected sex and tattoos, which transmit AIDS.	0.818					
It is easy for me to look after my individual sanitation such as washing after each bowel movement during menstruation	0.809					
The decision to reduce the consumption of fast food is out of my control.	0.808					
Although I have nausea with iron supplements, I try to always use it with food.	0.806					
Despite having little time, consumption of iron-rich foods (such as meat, liver, legumes such as lentils, etc.) is possible for me.	0.793					
Despite the pleasure of eating fast food with friends, it is easy for me to have homemade and fresh food.	0.790					
I’m sure that I can manage mental and physical signs of adolescence period.	0.785					
I am sure that I can consume enough fruit (2 to 4 servings) daily.	0.777					
I can behave normally with people who are HIV-positive.	0.767					
I am sure that I can I take 2 to 3 servings of dairy products daily.	0.761					
I’m sure that I can do proper exercise (such as walking, swimming, fitness, yoga, etc.) for at least 30 to 60 min per day and 5 d a week.	0.741					
The decision to do proper exercise (such as walking, swimming, fitness, yoga, etc.) for at least 30 to 60 min per day and 5 d a week is out of my control.	0.739					
I’m sure that I can have my homemade and fresh food.	0.725					
Most people who are important to me think that I should take shower standing during menstruation.		0.894				
My family believes that I should continue my social activities during menstruation.		0.865				
My family believes that I should have my social activities during adolescence similar to my other life periods.		0.860				
I feel that I am under pressure from those around me for doing physical activity.		0.856				
The people around me expect me to have a proper diet (adequate intake of fruit and fresh vegetables, dairy, etc.) During my puberty.		0.851				
The people around me expect to keep your weight balanced.		0.851				
The people around me expect me to keep my weight balanced.		0.850				
Most people who are important to me want me to have a proper diet (adequate intake of fruit and fresh vegetables, dairy, etc.) during my puberty.		0.849				
Most people who are important to me think that I need to do physical activity for 30 to 60 min per day and 5 d a week.		0.839				
People around me think that I should avoid risky behaviors (unprotected sex, injection of contaminated blood, and tattoos which result in AIDS.		0.833				
People who are important to me ask me to do physical activity for 30 to 60 min per day and 5 d a week.		0.830				
Most people who are important to me want me to avoid risky behaviors which lead to HIV infection.		0.823				
Most people who are important to me expect me to avoid risky behaviors that lead to HIV infection.		0.820				
Most people who are important to me think that I should follow a proper diet in puberty.		0.813				
I am under pressure from my surrounding people to have a healthy diet.		0.804				
The people surrounding me want me to look after my personal sanitation (bathing, changing underwear, etc.) during my puberty in order to prevent any infection.		0.802				
The people around me expect me to do daily physical activity for 30 to 60 min per day and 5 d a week.		0.775				
Most of my friends and classmates approve my unwillingness in doing my homework due to changes in my mood as well as my depression because of my menstruation.		0.669				
Most people who are important to me think that the changes during puberty such as physical and emotional changes prevent me from carrying out social activities such as attending social meetings.		0.660				
Consumption of iron-rich foods (such as meat, cereals, and dried fruits like apricot) in order to prevent from anemia is quite a useful behavior among women.			0.844			
Daily consumption of 2 to 4 servings of fruit is quite a useful behavior.			0.839			
Proper washing (first wash the vagina and then the anus) after each bowel movement in the toilet in order to prevent Pelvic Inflammatory Disease is quite an important behavior among women.			0.829			
Changing underwear daily in order to prevent uterine infection is quite an important behavior in women.			0.826			
Doing some exercise (such as walking smoothly, swimming, fitness, yoga, etc.) for at least 30 to 60 min per day and 5 d a week is quite a useful behavior.			0.822			
Taking shower standing, especially during periods is quite an important behavior.			0.815			
In my opinion, AIDS is a serious problem for the health of all people.			0.811			
In my opinion, personal hygiene during menstruation (such as washing genitals and anal area after each excretion and defecation) is essential.			0.788			
In my opinion, individual health during menstruation prevents the risk of infection.			0.776			
Having a proper diet (including fresh fruit and vegetables, dairy, etc.) is quite useful behavior during puberty.			0.769			
In my opinion, people with AIDS should inform others about their condition.			0.755			
In my opinion, puberty decreases the interest in daily activities such as loss of interest in activities within home.			0.751			
In my opinion, oversleeping during puberty results in impatience in doing homework.			0.736			
If my friend gets AIDS, I will cut my relationship with her.			0.732			
If a family member gets AIDS, he/she should be left alone.			0.730			
People with AIDS should be kept away from school.			0.721			
In my opinion, puberty causes a sharp and aggressive behavior in dealing with others.			0.718			
Excess in junk food consumption during menstruation is absolutely a useless behavior.			0.674			
In my opinion, menstruation disturbs the everyday life activities.			0.606			
In my opinion, menstruation creates difficulties in concentration on some activities such as studying a lesson.			0.570			
In my opinion, menstruation decreases the interest in doing school activities.			0.563			
I expect to have a good diet.				0.877		
I want to have a good diet.				0.841		
Within next three months, I’m going to avoid risky behaviors (such as transfusions of infected blood, unprotected sex, etc.) that lead to HIV infection.				0.836		
I have decided to frequently change my menstrual pad during menstruation.				0.826		
I want to do physical activity for 30 to 60 min per day and 5 d a week.				0.826		
I expect to have a proper diet.				0.815		
I currently, more or less, follow a proper diet.				0.810		
I’ve planned to have a proper diet.				0.809		
I have planned to avoid risky behaviors(such as transfusions of infected blood, unprotected sex, etc.) that lead to HIV infection				0.808		
I want to follow health issues for puberty period (such as bathing, changing underwear, etc.).				0.807		
I have planned to follow health issues for puberty period (such as bathing, changing underwear, etc.).				0.806		
I expect to do physical activity for 30 to 60 min per day and 5 d a week.				0.796		
I’ve planned to do physical activity for 30 to 60 min per day and 5 d a week.				0.782		
I have decided to continue my social activities during menstruation similar to my former normal life activities.				0.779		
I’m going to wash after each bowel movement during menstruation.				0.770		
I’m going to do physical activity for 30 to 60 min per day and 5 d a week.				0.764		
I’m going to follow health issues for puberty period (such as bathing, changing underwear, etc.).				0.694		
I do exercise at least 30 to 60 min per day.					0.873	
During my puberty, I change my underwear daily.					0.853	
I take standing bath, especially during my menstruation.					0.838	
I follow puberty period sanitation issues, especially individual sanitation.					0.827	
After each bowel movement, I wash uterus area properly from front to back (first washing the vagina and then the anus) in order to be clean and prevent Pelvic Inflammatory Disease.					0.822	
I don’t go to sea and pool during my menstruation.					0.814	
I avoid having contact with sharp objects such as HIV contaminated syringes, and needles infected with HIV.					0.814	
I avoid drinking drinks which have caffeine (such as strong black tea, espresso…) in order to prevent pre-menstrual symptoms (e.g., nervousness and menstrual pain, etc.).					0.805	
I consume 2 to 4 servings of fruit daily.					0.792	
I avoid doing risky behaviors such as unprotected sex and tattoo which transmit HIV.					0.773	
I consume 3 to 5 servings of vegetables daily.					0.772	
I would use cotton underclothes during puberty.					0.750	
During my puberty, I do daily physical activities which reduce my depression and aggression.					0.719	
I do exercise at least 30 to 60 min per day.					0.873	
During my puberty, I change my underwear daily.					0.853	
My parents provide a suitable nutrition for me during my puberty.						0.925
My parents determine how much I should read on the subject of nutrition.						0.905
My parents give me enough training and guidance on the subject of AIDS.						0.895
My parents determine how much I should read on the subject of adolescent health.						0.890
My parents give me enough training and guidance on the subject of adolescent health.						0.885
My parents determine how much I should read on the subject of AIDS.						0.878
My parents give me enough training and guidance about menstrual hygiene.						0.856
My parents determine how much I should read about menstrual hygiene.						0.851
Eigenvalue	17.15	13.06	12.09	11.95	8.78	6.53
Explained Variance (%)	16.49	12.56	11.62	11.49	8.44	6.28
Cumulative Variance (%)	16.49	29.05	40.68	52.17	60.62	66.90

CFA results confirmed the exploratory six-factor structure (RMSEA, χ^2^/df, TLI, IFI, NFI, CFI, AGFI, GFI and SRMR) (Table 4).

**Table 4:** Goodness of fit indexes for reproductive health dimensions (n =289)

***Construct***	***RMSEA***	***LO_90_***	***HI_90_***	***χ^2^/df***	**TLI**	**IFI**	**NFI**	**CFI**	**AGFI**	**GFI**	**SRMR**
Summary of rules of thumb	<0.5*			0.00	>0.8			>0.9			<0.6/
	<0.1**										<4
Nutrition and Exercise	0.082	0.079	0.086	2.94499	0.854	0.863	0.807	0.863	0.686	0.718	0.020
Adolescents’ health	0.057	0.047	0.065	1.91181	0.949	0.957	0.913	0.956	0.866	0.0896	0.018
Menstrual health	0.059	0.049	0.068	1.98328	0.950	0.958	0.920	0.958	0.876	0.905	0.017
AIDS	0.056	0.067	0.046	1.95648	0.961	0.968	0.936	0.968	0.890	0.919	0.019

Df= Degrees of Freedom, SB Chi-Square = Satorra–Bentler chi-square, Prob = Probability

* Good fit,

** Mediocre fit

## Discussion

This study was prospected to develop and validate an instrument for assessing the adolescents’ reproductive health and behaviors. In this study, the constructs of the modified theory of TPB were evaluated using the direct method (24). EFA was conducted for TBP structures and it led to removal of 10 items from the original questionnaire. The final form with 104 items was classified into six subscales. Having both exploratory and factor analyses applied, the results indicated a good structure for this new instrument. Exploratory factor analysis indicated that the six-factor structure of the questionnaire could jointly account for 67% of the cumulative observed variance.

Moreover, according to the results of CFA, the questionnaire with 4 given domains is a good instrument for measuring the reproductive health among adolescents in Iran.

In this study, a ratio of 0.64 was calculated for the content validity and the mean of the content validity index (CVI= 0.74) was likewise calculated. The mean of the CVR and CVI was reasonable and satisfactory. However, they obtained low values in some questions rooted in differences in the cultural context of the countries, so they have amended again in writing.

Results of the internal consistency suggest that the provided questionnaire had acceptable reliability. The study revealed an internal consistency of 0.85 to 0.91 for TPB (25). In addition, an internal consistency of 0.86 was reported for the constructs of the TPB (26).

Moreover, internal consistency of the final scale indicates a desirable reliability. In addition, ICC showed appropriate stability for the scale as it was examined by 45 participants with a 2-week interval (0.92) (15).

This instrument had a number of strengths. Adding item 28 to the instrument was one of them. Another important feature of our questionnaire lies in the way it was worked out; in spite of other questionnaires with short statements, we used long and complete sentences. The main feature of the BBRHQ was the fact that it was developed for middle age students and contained items on nutrition and exercise, adolescents’ health, menstrual health, and AIDS. Notably, a previous study among adolescent boys aged 15–18 yr assessed their sexual and reproductive health knowledge, attitude, and behaviors in Tehran (13). The advantages of the current study over the previous instrument were that the current instrument covers other aspects of reproductive health such as nutrition, exercise, puberty, menstrual hygiene, and AIDS. Its psychometric aspects have also been evaluated while other previous instruments lack this point.

## Conclusion

The BBRHQ is a valid and reliable instrument for evaluating the reproductive health, attitude, and behavior among female adolescents in Iran.

## Ethical considerations

Ethical issues (Including plagiarism, informed consent, misconduct, data fabrication and/or falsification, double publication and/or submission, redundancy, etc.) have been completely observed by the authors.

## References

[1] Glasier A Gülmezoglu AM Schmid GP (2006). Sexual and reproductive health: a matter of life and death. Lancet, 368:1595–1607. 17084760 10.1016/S0140-6736(06)69478-6

[2] HIV/AIDS JUNPo (2004). 2004 report on the global AIDS epidemic: 4th global report. ed. Unaids. http://files.unaids.org/en/media/unaids/contentassets/documents/unaidspublication/2004/GAR2004_en.pdf

[3] Mays RM Sturm LA Zimet GD (2004). Parental perspectives on vaccinating children against sexually transmitted infections. Soc Sci Med, 58(7):1405–13. 14759685 10.1016/S0277-9536(03)00335-6

[4] Statistical Centre of Iran (2011). Population and housing census. 2011 ed https://www.amar.org.ir/Portals/1/Iran/90.pdf

[5] Sawyer SM Afifi RA Bearinger LH (2012). Adolescence: a foundation for future health. Lancet, 379:1630–1640. 22538178 10.1016/S0140-6736(12)60072-5

[6] Khalajabadi-Farahani F (2014). Methodological considerations in studying sexual behaviors of young people in Iran. J Reprod Infertil, 15:171–172. 25469325 PMC4227973

[7] Ajzen I Madden TJ (1986). Prediction of goal-directed behavior: Attitudes, intentions, and perceived behavioral control. J Exp Soc Psychol, 22:453–474.

[8] Christian J Armitage CJ Abrams D (2007). Evidence that theory of planned behaviour variables mediate the effects of socio-demographic variables on homeless people’s participation in service programmes. J Health Psychol, 12:805–817. 17855464 10.1177/1359105307080615

[9] Armitage CJ Norman P Conner M (2002). Can the Theory of Planned Behaviour mediate the effects of age, gender and multidimensional health locus of control? Br J Health Psychol, 7:299–316. 12614502 10.1348/135910702760213698

[10] Desrichard O Roché S Bègue L (2007). The theory of planned behavior as mediator of the effect of parental supervision: A study of intentions to violate driving rules in a representative sample of adolescents. J Safety Res, 38:447–452. 17884431 10.1016/j.jsr.2007.01.012

[11] Ajzen I (1991). The theory of planned behavior. Organ Behav Hum Decis Process, 50:179–211.

[12] Anderssen N Jacobs D Aas H Jakobsen R (1995). Do adolescents and parents report each other’s physical activity accurately? Scand J Med Sci Sports, 5:302–307. 8581574 10.1111/j.1600-0838.1995.tb00049.x

[13] Mohammadi MR Mohammad K Farahani FK (2006). Reproductive knowledge, attitudes and behavior among adolescent males in Tehran, Iran. Int Fam Plan Perspect, 32:35–44. 16723300 10.1363/3203506

[14] Karimy M Niknami S Heidarnia AR (2013). Prevalence and determinants of male adolescents’ smoking in Iran: An explanation based on the theory of planned behavior. Iran Red Crescent Med J, 15:187–193. 23983996 10.5812/ircmj.3378 PMC3745745

[15] Ajzen I (2006). Constructing a TpB questionnaire: conceptual and methodological considerations. https://pdfs.semanticscholar.org/0574/b20bd58130dd5a961f1a2db10fd1fcbae95d.pdf

[16] Cleland J Verrall J Vaessen M (1983). Preferences for the sex of children and their influence on reproductive behaviour. https://www.popline.org/node/401264

[17] Ajzen I (2011). The theory of planned behaviour: reactions and reflections. Psychol Health, 26:1113–1127. 21929476 10.1080/08870446.2011.613995

[18] Brown R Ogden J (2004). Children’s eating attitudes and behaviour: a study of the modelling and control theories of parental influence. Health Educ Res, 19:261–271. 15140846 10.1093/her/cyg040

[19] Lawshe CH (1975). A quantitative approach to content validity1. Pers Psychol, 28:563–575.

[20] Polit DF Beck CT (2006). The content validity index: are you sure you know what’s being reported? Critique and recommendations. Res Nurs Health, 29:489–497. 16977646 10.1002/nur.20147

[21] Sharma S (1995). Applied Multivariate Techniques. John Wiley & Sons, Inc New York, NY, USA.

[22] Schneider Z Whitehead D (2013). Nursing and midwifery research: Methods and appraisal for evidence-based practice. ed. Elsevier Australia.

[23] Koo TK Li MY (2016). A Guideline of Selecting and Reporting Intraclass Correlation Coefficients for Reliability Research. J Chiropr Med, 15:2155–163. 10.1016/j.jcm.2016.02.012 PMC4913118 27330520

[24] Francis JJ Eccles MP Johnston M (2004). Constructing questionnaires based on the theory of planned behaviour: A manual for health services researchers. http://openaccess.city.ac.uk/1735/1/TPB%20Manual%20FINAL%20May2004.pdf

[25] De Bourdeaudhuij I Klepp K Due P (2005). Reliability and validity of a questionnaire to measure personal, social and environmental correlates of fruit and vegetable intake in 10–11-year-old children in five European countries. Public Health Nutr, 8:189–200. 15877912 10.1079/phn2004673

[26] Diamond HC (2009). The role of gender in staying smoke-free in adolescence: Using a theory of planned behavior approach. University of Prince Edward Island, 2009 .

